# The removal of chromium (VI) from tannery waste using Spirulina sp. immobilized silica gel

**DOI:** 10.3906/kim-2106-22

**Published:** 2021-08-09

**Authors:** Nais Pinta ADETYA, Uma Fadzilia ARIFIN, Emiliana ANGGRIYANI

**Affiliations:** 1 Department of Leather Processing Technology, Politeknik ATK Yogyakarta, Sewon, Bantul, Yogyakarta, Indonesia; 2 Department of Rubber and Plastic Processing Technology, Politeknik ATK Yogyakarta, Sewon, Bantul, Yogyakarta, Indonesia

**Keywords:** Adsorption, chromium, immobilization, Spirulina sp., tannery waste

## Abstract

The use of microalgae biomass is an alternative solution to the problem of environmental pollution due to heavy metals, one of which is Cr metal in leather tanning liquid waste. However, the ability of biomass to adsorb heavy metals has limitations. Therefore, the algal biomass is immobilized with silica gel in order to obtain a stable structure. This research aims to study the absorption efficiency of Cr (VI) metal by the biomass of Spirulina sp. which is immobilized with silica gel from the tannery liquid waste. The preparation stages in this study were adsorbent preparation, immobilization of biomass with silica gel, and preparation of tannery liquid waste. Furthermore, the research treatment was carried out to determine the effect of the independent variable on the adsorption of Cr (VI). Characteristics of functional groups using FTIR show the biomass constituent of Spirulina sp. immobilized containing amino, carboxylate, and hydroxyl groups. The results showed that the optimum contact time required for adsorption of Cr (VI) ions was 60 min of immersion and the optimum pH value was 3. Adsorption of Cr (VI) ions followed Freundlich adsorption isothermal and included in the pseudo second order adsorption kinetics.

## 1. Introduction

The leather tanning industry is one of the industries that releases a large volume of liquid waste. In tanning 1 ton of wet leather, about 40 m^3^ of water is needed and then disposed of as liquid waste mixed with chemical residues of the process and leather components dissolved during tanning [1]. Conventional leather tanning using chrome as a tanning material has an impact on the environment because it brings the remaining chromium into the liquid waste. Although the chromium used in the tanning process is trivalent chromium, hexavalent chromium is always present in the liquid waste [2]. The disposal of chromium with liquid waste is hazardous and toxic contamination, because chromium is a type of heavy metal waste that is difficult to decompose and can accumulate in the body and the environment [3].

Chrome (Cr) is the most widely used tanner in the leather tanning industry and about 85% of the world’s leather is tanned using chrome. This is based on the fact that chromium is able to react and form bonds with skin collagen protein amino acids [4]. Cr (IV) is a heavy metal that is toxic, and its toxicity depends on the valence of the ion, and the toxicity of Cr (IV) is about 100 times the toxicity of Cr (III) [5,6]. In addition, Cr (VI) is highly corrosive and carcinogenic. Cr (III) is a nutrient that the human body needs in the amount of about 50–200 μg/day. However, it is feared that in an alkaline environment and the presence of certain oxidizers or certain conditions it is possible for Cr (III) ions to be oxidized to Cr (VI) [7]. Therefore, the Cr metal in the tannery industry liquid waste needs to be handled first before being discharged into water bodies or rivers.

Several species of microalgae have been found to have the potential to adsorb metal ions, both in the living (active) state and in the form of dead cells (inactive biomass) [8]. The use of microalgae biomass for adsorbents aims to reduce the use of nonrenewable inorganic flocculants and synthetic flocculants that are not easily biodegradable [9]. Spirulina sp. is known to be able to adsorb metal ions because there are functional groups in microalgae that can bind with metal ions. These functional groups are carboxyl, hydroxyl, amino, sulfate, and sulfonate groups that are present in the cell wall in the cytoplasm [10].

However, the ability of microalgae to absorb metal ions is very limited by several disadvantages such as very small size, low density, and the algae is easily damaged due to degradation by other microorganisms [11]. To overcome these weaknesses, biomass immobilization was carried out with silica gel. By immobilizing the algae, the adsorbent size will be larger and have a stable aggregate form [12]. This research studied the optimum pH conditions, contact time, and metal ion concentration used in the metal ion adsorption process, as well as the immobilization effect on metal ion absorption and its application in reducing Cr (VI) levels in leather tanning wastewater.

## 2. Materials and methods

This research was conducted in two steps. First is the preparation of biomass and immobilization of biomass on silica gel. The second is to determine the effect of Cr ion absorption by adsorbent with variations in the pH value of the metal solution, the contact time, and the concentration of the metal solution.

### 2.1. Materials

The materials used in this study were as follows: Spirulina platensis biomass from CV. Neoalgae (Sukoharjo, Indonesia), a sample of chromium tanning process waste from a leather tanning industry in Magetan, Na_2_SiO_3_ (Merck, 99.5%), HCl (Merck, 99.5%), whatman 41 filter paper, and 1000 mg/L pure chrome standard solution from K_2_Cr_2_O_7_ (Merck, 99.5%).

### 2.2. Biomass preparation and immobilization on silica gel

Cultivation of Spirulina sp. in Spirulina Media with growing conditions at 28 °C and lighting 3000 lx. After 14 days of cultivation, cells were harvested by centrifugation and washed several times to remove the culture medium and filtered with whatman 41 filter paper to reduce the water content. Biomass was dried in an oven at 60 °C for 24 h until dry, then mashed with a mortar and sieved to a size of 40 mesh [13]. The immobilization process was carried out by taking 100 mL of sodium silicate Na_2_SiO_3_ solution and dropping it with concentrated HCl to pH = 7. The mixture was stirred until aqua-gel (hydrogel) was obtained, and 3 grams of microalgae biomass was added. Then dried in an oven at a temperature of 80 °C to form dry silica [14]. Characterization of functional groups was carried out on the biomass before and after the adsorption process by FTIR.

### 2.3. Preparation of Cr (VI) stock solution

Cr (VI) stock solution 1000 mg/L solution was prepared by dissolving 2.8288 g of potassium dichromate (K_2_Cr_2_O_7_) powder with distilled water up to a volume of 1000 mL.

### 2.4. Effect of pH variations

Biomass is mixed with a metal ion solution which has a certain concentration, adjusting the pH value using 0.1 M nitric acid solution and 0.1 M ammonia solution. Each 100 mL metal ion solution with a concentration of 20 mg/L pH set to 2, 3, 4, and 5. Each solution was mixed with 400 mg of biomass in 250 mL erlenmeyer flask for 60 min and stirred with a magnetic stirrer 120 rpm at room temperature. The supernatant was filtered with whatman 41 filter paper.

### 2.5. Effects of contact time and initial concentration

100 mL metal solution with a concentration of 20 mg/L at optimum pH value was mixed with biomass in 250 mL erlenmeyer and stirred with a magnetic stirrer at room temperature, the contact time was varied to 30, 60, 90, 120, and 150 min. To determine the adsorption rate of Cr (VI), the initial concentrations (10, 20, 30, and 40 mg/L) were varied at the optimum pH value in a 250 mL erlenmeyer flask at room temperature during the optimum time. The biomass dose was kept constant at 400 mg and the stirring speed was 120 rpm. The concentration of Cr (VI) ion after adsorption was determined using the spectrophotometric method.

### 2.6. Adsorption isotherms

Adsorption isotherm is a function of the concentration of solute adsorbed on the solid to the solution concentration. Determination of the adsorption isotherm using the initial concentration range of 10, 20, 30, and 40 mg/L and carried out for 60 min. The adsorption capacity of an adsorbent for a contaminant can be determined by calculating the adsorption isotherm. The adsorption isotherm shows an equilibrium relationship between the adsorbate concentration in the fluid and the adsorbent surface at a constant temperature. To test the data link between the adsorbent and the equilibrium concentration in the adsorption isothermal model was used that model of Langmuir and Freundlich isothermal [15]. The adsorption capacity can be calculated using equation 1 [13]:


(1) $$q_{e}=\frac{(C_{0}-C_{e})V}{m}$$

While removal of Cr (VI) (%) can be calculated using equation 2 [8]:


(2) $$\%\;removal\;of\;Cr\;(VI)\;ions=\frac{\left(C_{0}-C_{e}\right)}{C_{0}}\times 100$$

where **q_e_** is the biomass adsorption equilibrium ions uptake capacity (mg/g), **C_o_** is the initial ion concentration (mg/L), **C_e_** is the equilibrium or final ion concentration (mg/L), V is the volume of metal ion solution (L) and **m** is the Spirulina sp. biomass immobilized by silica gel dry weight (g).

Langmuir isothermal assumes the adsorption of a single layer on the surface containing a certain amount of adsorption centers with uniform adsorption energies without displacement of the adsorbate on the surface plane. The linear form of the Langmuir isothermal equation is shown in equation 3 [6]:


(3) $$\frac{C_{e}}{q_{e}}=\frac{1}{Q_{o}k_{L}}+\frac{C_{e}}{Q_{o}}$$

where, **Q_o_** is the maximum adsorption capacity (mg/g) and **K_L_** is the langmuir constant (L/mg).

The Freundlich isotherm is most commonly used because it is considered to be better at characterizing the adsorption process [16]. Freundlich isothermal is used at heterogeneous surface energies with different concentrations. The linear form of the Freundlich isotherm is shown by equation 4 [6]:


(4) $$\log(q_{e})=\log\;k_{F}+\frac{1}{n}\;\log\;C_{e}$$

where **K_f_** is the adsorption capacity at unit concentration (mg/g); **n** is the intensity of adsorption.

### 2.7. Adsorption rate kinetics

Determination of the adsorption kinetics model in this study was carried out by varying the absorption time from 30, 60, 90, 120, 150 min at pH value 3 with a concentration of Cr(VI) 20 mg/L. Pseudo first and second order kinetics equations were used to determine the adsorption kinetics order. The determination of pseudo first order kinetics can use the following equation 5 [6]:


(5) $$\ln(q_{e}-q_{t})=\ln\;q_{e}-k_{1t}$$

While the determination of pseudo second order kinetics can use the following equation 6 [13]:


(6) $$\frac{t}{q_{t}}=\frac{1}{k_{2}{q_{e}}^{2}}+\frac{1}{q_{e}}\;t$$

where **q_t_** is the amount of adsorbate adsorbed (mg/g) at time t, **q_e_** is the amount of adsorbate adsorbed (mg/g) at the best time (0 to t < **q_e_**) and k is the adsorption rate constant.

### 2.8. Cr (VI) removal from tanning waste industry

Chrome tanning waste is filtered using technical filter paper to remove impurities. The filtrate is used as a sample of liquid waste. As a characterization step, the sample was analyzed to determine the level of Cr (VI), and the results of the analysis were the initial concentration of chromium in wastewater. The final concentration of Cr in the waste was determined by adding 1 gram of Spirulina sp. into 100 mL of liquid waste with optimum pH and stirred with a magnetic stirrer at room temperature for the optimum time. Then filtered with whatman 41 filter paper. The resulting filtrate was analyzed for the Cr (VI) content as the final Cr (VI) content in the waste, while the biomass was characterized using FTIR.

## 3. Results and discussion

### 3.1. FTIR characteristics of Spirulina sp. immobilized silica gel

**Figure 1:**
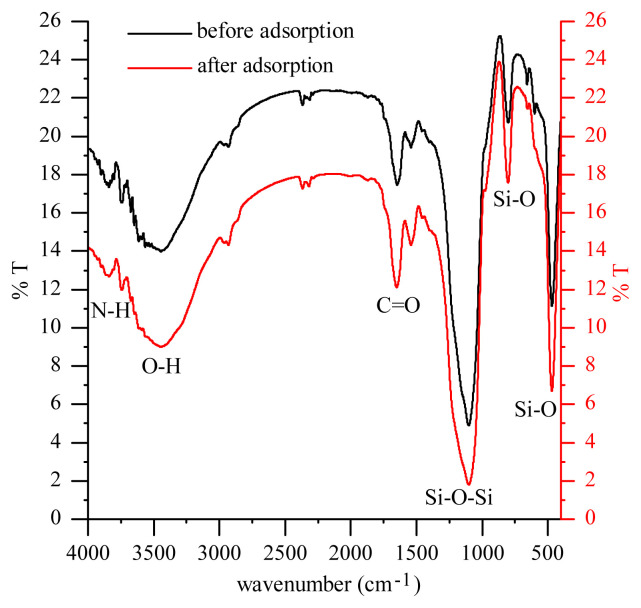
FTIR spectrum of Spirulina sp. immobilized before and after adsorption of Cr (VI).

The results of identification of the functional groups of Spirulina sp. immobilized before and after interaction with the Cr (VI) metal ion is shown in Figure 1. Based on the FTIR spectrum, the biomass of Spirulina sp. before the interaction with the Cr (VI) metal ion, it appears that the absorption of the medium around the wave number 3747.95 cm^−1^ is the absorption of the OH-alcohol stretching vibration. The width absorption is around the wave number 3445.95 cm^−1^, which is the stretching vibration absorption from the primary N-H group and the absorption around the wave number 2365.19 cm^−1^. This absorption indicates a C-H stretching vibration. The absorption band around the wave number 1648.05 cm^−1^ indicates a stretching vibration of C = O (carboxylate-ester). The absorption around the wave number 1102.24 cm^−1^ indicates an asymmetrical stretching vibration of Si-O. The absorption band around the wave number 468.84 cm^−1^ showed the presence of Si-O stretching vibrations from Si-O-Si, which was obtained from the immobilization of biomass with silica gel [17].

Based on the FTIR spectrum of Spirulina sp. immobilized after interaction with the metal ion Cr (VI), a medium absorption band appears around the wave number 3742.27 cm^−1^, which is the absorption of the stretching vibration of OH-alcohol. The presence of sharp absorption around the wave number 3445.81 cm^−1^ is the absorption width of the stretching vibration of the primary N-H group and the presence of absorption around the wave number 2366.95 cm^−1^ indicates C-H stretching vibrations. The absorption band around the wave number 1650.01 cm^−1^ indicates a stretching vibration of C = O (carboxylate, ester). The presence of strong absorption around the wave number 469.46 cm^−1^ was identified as the stretching vibration of Si-O from Si-O-Si. The functional groups that experience a shift in wave numbers are assumed to be functional groups that may affect the adsorption process [18].

The process of immobilization of microalgae with sodium silicate solution was carried out using the sol gel method, namely the addition of HCl. The addition of concentrated HCl solution is intended for the process of forming free silicic acid, which can bind to form dimers, trimers and so on through a polycondensation reaction and the release of H_2_O molecules.

### 3.2. Effect of pH value on the Cr (VI) adsorption

**Figure 2:**
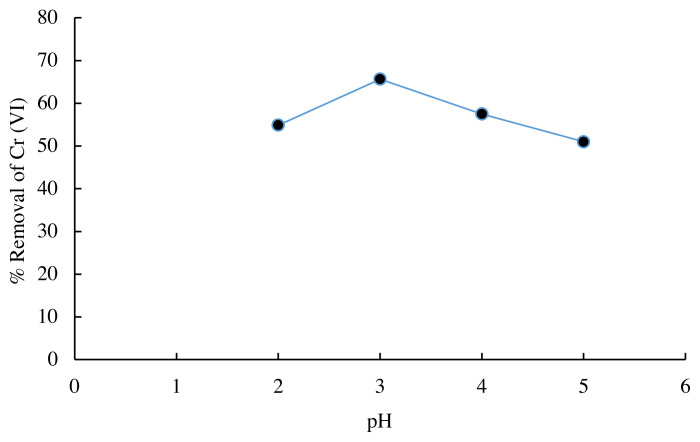
Effect of pH value on the Cr (VI) removal (biomass mass = 400 mg; initial Cr ion concentration = 20 mg/L; agitation speed=120 rpm; contact time = 60 min at room temperature).

**Figure 3:**
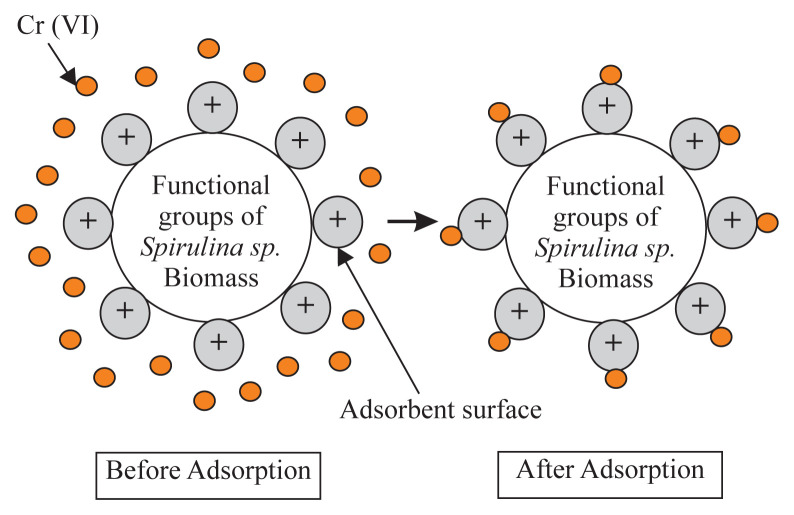
Mechanism of Cr (VI) adsorption on adsorbent.

The initial pH value of the solution is important for Cr (VI) adsorption because the protonation of the adsorbent configures the active ion exchange site and surface activity [19]. The results of the adsorption of Cr (VI) metal ions with pH variations are shown in Figure 2. The highest percentage of adsorption of Cr (VI) metal ions was achieved when the initial pH value of the solution was at pH = 3. The results of this study are close to the results of Pradhan et al. [6], which obtained the maximum pH absorption of Cr (VI) metal ions by Scenedesmus sp. at a pH = 2.65. At low pH value, the surface of the biomass containing anionic groups such as amines, carboxyl, and hydroxyl is protonated and becomes positively charged (Figure 3). At the same time, through its lone pair, the metal ion Cr (VI), which is present in the acid solution in the form of anionic species, such as tetraoxohydrochromate (HCrO_4_^−^), chromate (CrO_4_^2−^) and dichromate (Cr_2_O_7_^2−^), relatively easily interacts with the adsorbent, so that adsorbed metal ions are relatively large. The positively charged biomass surface attracts the anionic Cr (VI) species electrostatically, resulting in strong Cr (VI) physisorption to the biomass at lower pH value ranges [20]. Cr VI can form complexes with the NH_2_ functional group on Spirulina sp. biomass on an acidified surface by the following reaction:


(7) $${\text{HCrO}}_{4}^{-}+\text{R}-{\text{NH}}_{2}+{\text{H}}^{+}↔\text{R}-{\text{NH}}_{3}^{+}-{\text{HCrO}}_{4}^{-}$$

When the pH value of the solution increases gradually, the biomass surface becomes negatively charged due to the decrease in proton concentration. Negatively charged biomass competes with anionic chromate ions due to electrostatic repulsion, which results in decreased adsorption efficiency at higher pH value ranges [21].

### 3.3. Effect of contact time on Cr (VI) adsorption

**Figure 4:**
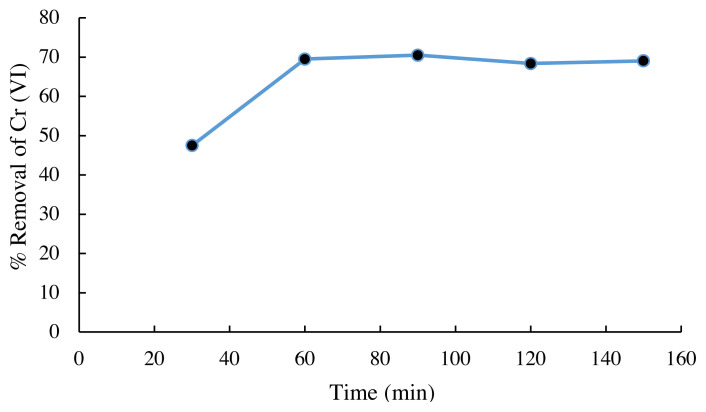
Effect of contact time on the Cr (VI) removal (biomass mass = 400 mg; pH = 3; initial Cr ion concentration = 20 mg/L; agitation speed=120 rpm at room temperature).

Figure 4 shows that in the initial minutes of interaction, the adsorption progresses faster because the number of active sites in the adsorbent is still quite a lot, after the adsorption process lasts for 60 min, the adsorption is relatively constant. In accordance with the theory, the adsorption process that does not depend on metabolic processes or the absorption process of metal ions, which only occurs on the surface of the cell wall, takes place relatively quickly because it does not involve the process of metal accumulation in cells [19]. The addition of time up to 150 min did not significantly increase the amount of Cr (VI) absorbed. In this state it can be considered that an equilibrium has been reached where all the active sites on the adsorbent Spirulina sp. immobilized on saturated silica gel, or all active sites have been filled with Cr (VI) ions. After equilibrium is achieved, the amount of metal ions absorbed does not change significantly with the addition of contact time between the Cr (VI) metal ion and the adsorbent [20].

### 3.4. Effect of initial concentration of metal solutions on Cr (VI) adsorption

**Figure 5:**
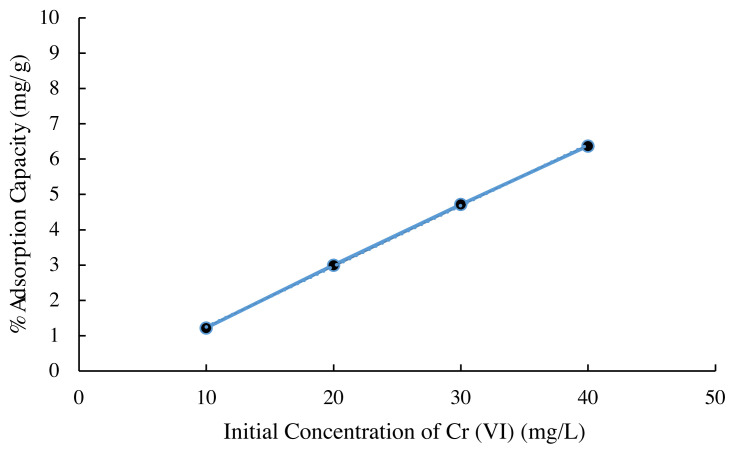
Effect of initial concentration on the removal of Cr (VI) (biomass mass = 400 mg; pH = 3; agitation speed=120 rpm; contact time = 60 min at room temperature).

Figure 5 shows that the amount of chromium absorbed by the biomass is influenced by variations in the concentration of the solution used. The greater the concentration of the solution interacted with the fixed amount of adsorbent, the greater the amount of Cr (VI) absorbed by the adsorbent. In accordance with Langmuir’s theory, which states that on the surface of the absorbent, in this case the adsorbent Spirulina sp., there is a certain number of active sites, which are proportional to the surface area of the absorber. So that, as long as the active site is not saturated or in a balanced state, the increasing concentration of metal ions being contacted will also increase the amount of metal ions that are absorbed [22]. However, the biomass surface has a limited binding site, after completing the adsorption at the site, further loading of Cr is not possible [13]. Furthermore, the data on the variation in the concentration of Cr (VI) metal ions can be used to find the adsorption isotherm of Cr (VI) metal ions by biomass.

### 3.5. Adsorption isotherm model

**Figure 6:**
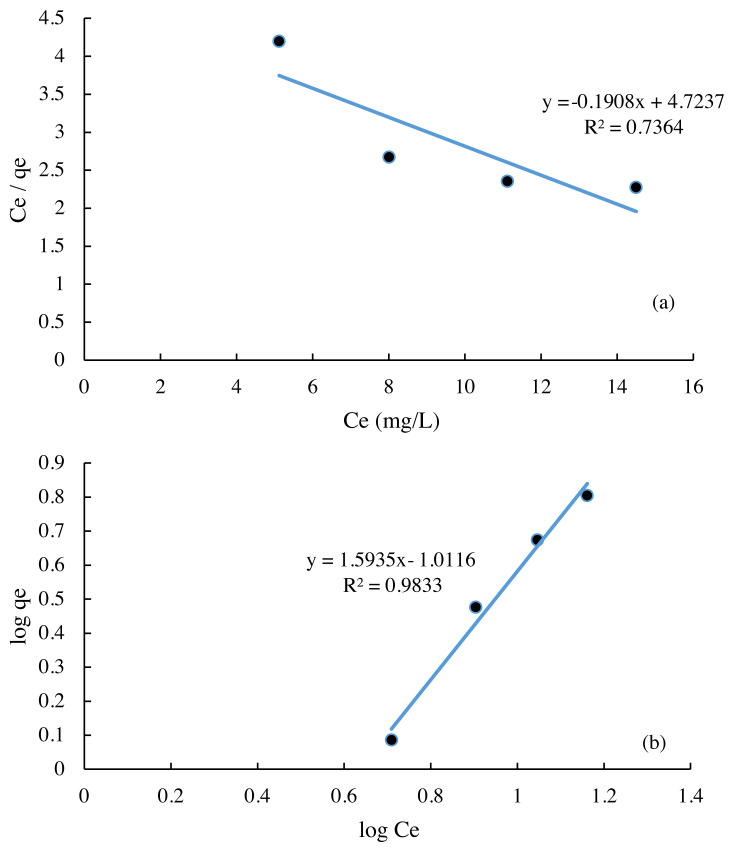
Adsorption isotherm (a) Langmuir and (b) Freundlich isotherm for Cr (VI) adsorption.

The data used to find the adsorption isotherm is the absorption data on the variation in the concentration of Cr (VI) metal ions used by the biomass of Spirulina sp. immobilized on the silica gel. Langmuir isotherm explains that on the surface of the absorber, in this case the biomass of Spirulina sp., there is a certain number of active sites, which are proportional to the surface area of the absorber. Each active site has the same energy, so that it can be said that the adsorbent surface is homogeneous. Whereas Freundlich’s adsorption isotherm states that the adsorbent surface is heterogeneous, this means that the affinity of each active center is not the same, so that adsorption on the most active site is preferred [6]. The results of processing data on variations in the concentration of Cr (VI) metal ions used to find Langmuir adsorption isotherms and Friendlich isotherms are presented in Figure 6.

Based on the R2 value, it can be assumed that the Freundlich isotherm (R2 = 0.9833) can interpret the adsorption data better than the Langmuir isotherm (R2 = 0.7364). This suggests that the surface possibility of Spirulina sp. used is heterogeneous, meaning that each active site in a complex algal matrix has different energies or affinities. In addition, these data indicate that the adsorption mechanism occurs physically so that the bond between the biomass adsorbent Spirulina sp. with the adsorbate is weak and forms a multilayer layer. This weak bond is expected to be easy to desorption so that the adsorbent can be reused [20].

Table 1 shows the parameter values in the adsorption isotherm. The **k_f_** and 1/n values respectively indicate the adsorption capacity and adsorption intensity. The **k_f_** and **Q_o_** values are the maximum amount of adsorbate that can be absorbed by the adsorbent in mg. The greater the **k_f_** and **Q_o_** values, the greater the adsorption capacity. Based on these data, the biomass adsorbent of Spirulina sp. immobilized had a maximum adsorption capacity of 0.389 mg/g. The magnitude of 1/n gives the adsorption favorability measure. A value of 1/n between 1 and 10 indicates favorable uptake [23]. For this study, a value of 1/n also presented the same result, representing favorable uptake.

### 3.6. Adsorption kinetics model

Determination of the adsorption rate can be done through a kinetics model approach. These results can be interpreted through adsorption kinetics using two kinetics models, namely pseudo first order and pseudo second order kinetics models. Apart from many alternative models, pseudo first order and pseudo second order remain the most common models for batch processing to evaluate the control mechanism in adsorption systems [24].

**Figure 7:**
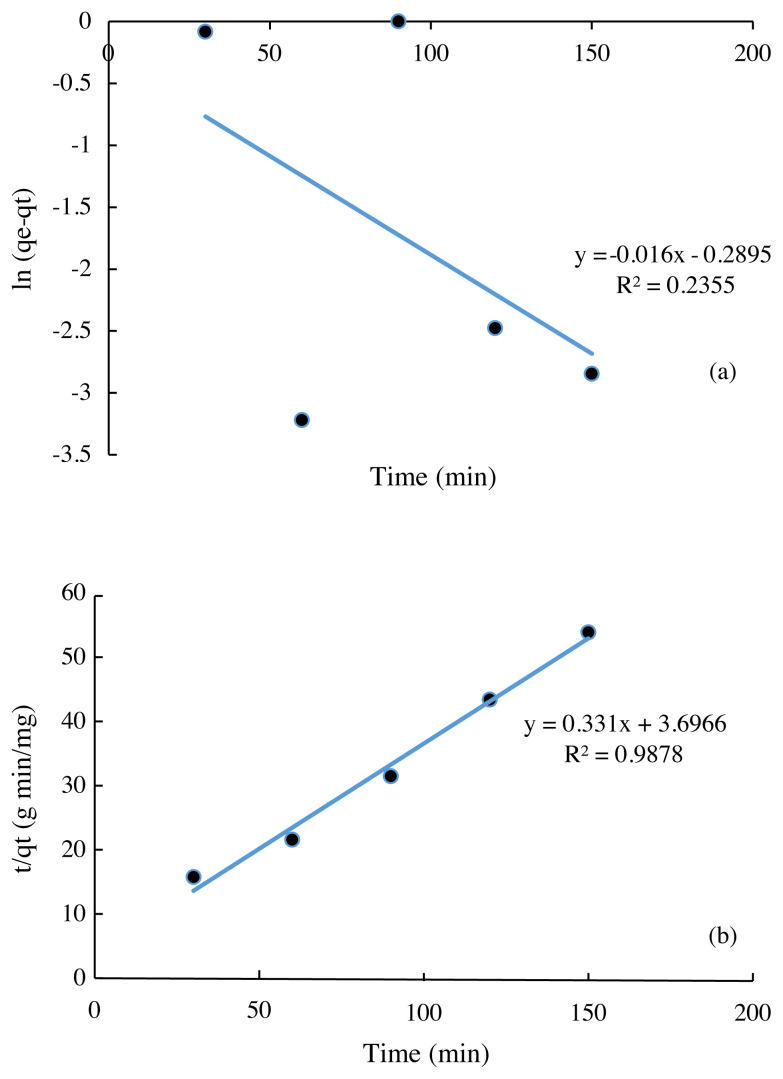
(a) Pseudo first order and (b) Pseudo second order Cr (VI) adsorption by Spirulina sp. immobilized silica gel.

The results in Figure 7 show that the pseudo-second order kinetics represent the adsorption rate kinetics in this experiment. This can be seen from the coefficient of determination and the value of q_e_. Table 2 shows that the pseudo-second order R^2^ value is closer to 1 (R^2^ = 0.9878) and is higher than the pseudo-first order (R^2^ = 0.2355). In addition, the calculated q_e_ value for pseudo second order kinetics of 3.0211 g/mg is closer to the experimental q_e_ (2.820 mg/g). The results showed that the pseudo-second order adsorption mechanism was dominant. The rate of the adsorption process is controlled by sharing or exchanging electrons between sorbent and sorbate [25].

**Figure 8:**
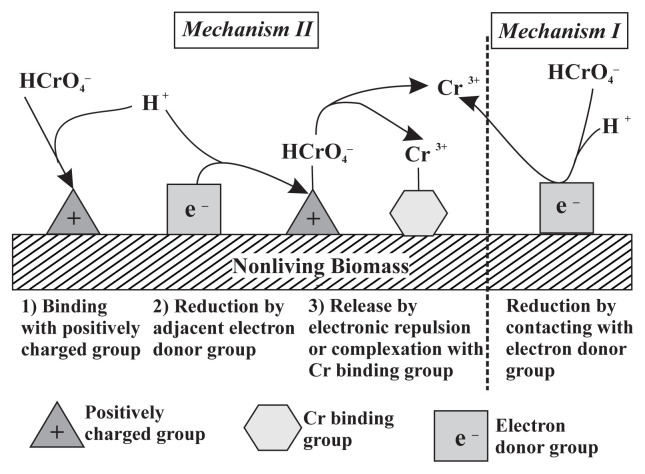
Proposed mechanism of Cr(VI) adsorption by nonliving biomass [26].

Removal of Cr (VI) by nonliving biomass occurs through two mechanisms, namely direct reduction and indirect reduction are presented in Figure 8 [26]. Based on this mechanism, Cr (VI) is reduced to Cr (III) by biomass in an acidic environment. Then some of the Cr (III) is adsorbed onto the biomass. The amount of adsorption depends on the nature of the biomass [27].

Spirulina sp. can adsorb Cr (VI) under live or nonliving conditions. When Spirulina sp. is incubated in Zarrouk’s medium in a flask containing Cr (VI) (as K_2_Cr_2_O_7_), the ion concentration in the medium decreased gradually, and a maximum of 60% was observed on the 7th day of incubation [28].

The comparison of adsorption capacity and removal efficiency of Cr (VI) of various algae biomass is shown in Table 3. The maximum Cr(VI) removal efficiency of dry Spirulina sp was 72% [28]. It is known that the percent adsorption of Cr (VI) by the biomass of Spirulina sp. immobilized in this study was lower than the uptake of metal ions Cr (VI) by the biomass of Spirulina sp. that is not immobilized. This is due to the presence of silica, which is covalently bound to the functional groups present in the biomass, causing a reduction in the active sites at Spirulina sp. immobilized. However, it is still recommended to immobilize the biomass because it can produce adsorbents that have good particle strength, porosity, high chemical resistance, resistance to the decomposition of other microorganisms. In addition, the adsorbent can be washed for reuse [11]. Spirulina sp. immobilized on silica gel was also found to efficiently degrade cadmium content from aquatic systems [14].

### 3.7. Cr (VI) removal from tanning waste industry

The metal content of Cr (VI) in the waste sample before the adsorption process was 0.0036 mg / L. For the absorption of this waste sample, 1 g of Spirulina sp. biomass adsorbent was used to adsorb the chromium metal present in the waste sample. The sample is then stirred with a stirrer for the optimum time. After stirring, the sample is filtered, and the filtrate is analyzed for its Cr (VI) content. The analysis showed that the chromium metal contained in the waste samples could not be detected (<0.0014 mg/L).

The adsorption percentage of Cr (VI) by Spirulina sp. immobilized in this study was lower than the uptake of Cr (VI) metal ions by Spirulina sp. who are not immobilized [13]. This is due to the presence of silica that binds covalently with the functional groups present in the biomass, causing a reduction in the active site in the biomass of Spirulina sp. who are immobilized. Although the adsorption capacity of immobilized biomass is lower, efforts to immobilize biomass are still recommended because, in addition to producing an adsorbent that has good particle strength, porosity and high chemical resistance, it is also resistant to the decomposition of other microorganisms, and the adsorbent can be washed for reuse [11].

## 4. Conclusion

Based on the research that has been done regarding the adsorption of Cr (VI) metal ions from tanning waste by the biomass of Spirulina sp. immobilized by silica gel, it can be concluded that the biomass of Spirulina sp. immobilized silica gel was found to be feasible in the adsorption of Cr (VI) from solution. FTIR spectrum of macromolecules that make up the biomass of Spirulina sp. immobilized containing amino, carboxylate, and hydroxyl groups. Adsorption of Cr (VI) ions followed Freundlich adsorption isothermal and included in the pseudo second order adsorption kinetics. The initial concentration of Cr (VI) in the tanning waste was 0.0036 mg / L and decreased to undetectable (<0.0014 mg/L) after the adsorption process using Spirulina sp. biomass immobilized silica gel.

## Figures and Tables

**Table 1:** Langmuir and Freundlich adsorption isotherm parameters.

Freundlich Isotherm			Langmuir Isotherm		
k_f_ (mg/g)	1/n	R^2^	Q_0_ (mg/g)	k_L_ (L/mg)	R^2^
0.3895	1.5935	0.9833	5.2411	0.9013	0.7364

**Table 2:** Predicted kinetic parameters for removal of Cr(VI).

Pseudo First Order				Pseudo Second Order			
q_e exp_ (mg/g)	q_e_ (mg/g)	K_1_ (1/min)	R^2^	q_e exp_ (mg/g)	q_e_ (mg/g)	K_2_ (g/mg min)	R^2^
2.8200	0.9841	0.2895	0.2355	2.8200	3.0211	0.0296	0.9878

**Table 3:** Comparison of alga biomass as adsorbent of Cr (VI).

Algae	Sorption Capacity (mg/g) (a) or Removal Efficiency (%) (b)	Ref.
*Scenedesmus sp*. (dried biomass)	0.11 (a)	[6]
*Nannochlorosis oculata* (dried biomass)	0.79 (a)	[29]
*Cladophora glomerata* (dried biomass)	0.19 (a) 63 (b)	[16]
*Enteromorpha intestinalis* (dried biomass)	0.10 (a) 47 (b)	[16]
*Microspora amoena* (dried biomass)	0.08 (a) 52 (b)	[16]
*Oscillatoria sp*. (dried biomass)	0.27 (a)	[30]
*Oscillatoria sp*. (immobilized with Ca-Alginate)	0.18 (a)	[30]
*Spirulina sp*. (dried biomass)	72 (b)	[28]
*Spirulina sp*. (immobilized with Na_2_SiO_3_)	0.39 (a) or 65 (b)	Present work
